# Water-stable, biocompatible, and highly luminescent perovskite nanocrystals-embedded fiber-based paper for anti-counterfeiting applications

**DOI:** 10.1186/s40580-023-00366-6

**Published:** 2023-05-03

**Authors:** Madhumita Patel, Rajkumar Patel, Chanho Park, Kanghee Cho, Pawan Kumar, Cheolmin Park, Won-Gun Koh

**Affiliations:** 1 Department of Chemical and Biomolecular Engineering, Yonsei University, 50 Yonsei-Ro, Seodaemun-Gu, Seoul, 120-749 South Korea; 2 Energy & Environmental Science and Engineering (EESE), Integrated Science and Engineering Division (ISED), Underwood International College, Yonsei University, 85 Songdogwahak-Ro, Yeonsu-Gu, Incheon, 21983 South Korea; 3 Department of Materials Science and Engineering, Yonsei University, 50 Yonsei-Ro, Seodaemun-Gu, Seoul, 120-749 South Korea; 4 Institute National de La Recherche Scientifique-Centre Énergie Materiaux Télecommunications (INRS-EMT), Varennes, QC Canada

**Keywords:** Perovskite nanocrystals, Electrospinning, Luminescent fiber, Pattern printing, Anti-counterfeiting

## Abstract

In this study, we present a promising and facile approach toward the fabrication of non-toxic, water-stable, and eco-friendly luminescent fiber paper composed of polycaprolactone (PCL) polymer and CsPbBr_3_@SiO_2_ core–shell perovskite nanocrystals. PCL-perovskite fiber paper was fabricated using a conventional electrospinning process. Transmission electron microscopy (TEM) clearly revealed incorporation of CsPbBr_3_@SiO_2_ nanocrystals in the fibers, while scanning electron microscopy (SEM) demonstrated that incorporation of CsPbBr_3_@SiO_2_ nanocrystals did not affect the surface and diameter of the PCL-perovskite fibers. In addition, thermogravimetric analysis (TGA) and contact angle measurements have demonstrated that the PCL-perovskite fibers exhibit excellent thermal and water stability. The fabricated PCL-perovskite fiber paper exhibited a bright green emission centered at 520 nm upon excitation by ultra-violet (UV) light (374 nm). We have demonstrated that fluorescent PCL-perovskite fiber paper is a promising candidate for anti-counterfeiting applications because various patterns can be printed on the paper, which only become visible after exposure to UV light at 365 nm. Cell proliferation tests revealed that the PCL-perovskite fibers are cytocompatibility. Consequently, they may be suitable for biocompatible anti-counterfeiting. The present study reveals that PCL-perovskite fibers may pave way toward next generation biomedical probe and anti-counterfeiting applications.

**Graphical Abstract:**

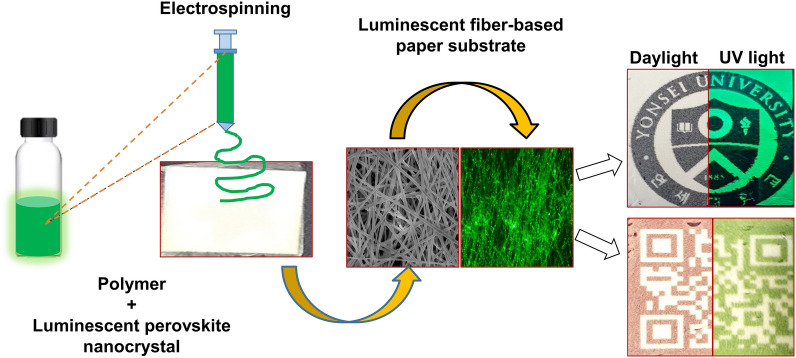

**Supplementary Information:**

The online version contains supplementary material available at 10.1186/s40580-023-00366-6.

## Introduction

Recently, all-inorganic perovskite CsPbX_3_ (X = Cl, Br, and I) nanocrystals have emerged as interesting materials for various application owing to their excellent optical and electrical properties, such as bio-imaging, drug delivery, photodetectors, laser devices, light-emitting diodes (LEDs), and photovoltaics [1–6]. Exceptional optical properties like bright photoluminescence, higher PLQY (photoluminescence quantum yield), high emission color purity, narrow emission spectra, tunable emission in the full visible-light region, and comparatively high photostability compared to organic dyes, as well as their low-cost synthesis, make all-inorganic perovskite CsPbX_3_ promising candidates for optoelectronics and bio-medical applications [7–9]. Therefore, all-inorganic perovskite CsPbX_3_ have been used in various applications over the past several years [10, 11]. Among all-inorganic perovskite, CsPbBr_3_ is considered one of the best choices and has been extensively explored in various applications due to its high PLQY and high stability when compared to CsPbCl_3_ and CsPbI_3_, and its organic–inorganic counterparts [8, 12].

Counterfeiting in various industries, such as goods, pharmaceuticals, currency, and security documents, is rapidly increasing with technological and socio-economical advancements [13, 14]. Therefore, counterfeiting has become a serious problem all over the world, including among consumers, governments, and industries. Consequently, researchers have devoted significant efforts to developing anti-counterfeiting techniques, such as holograms, radio frequency identification, plasmonic labels, quick response (QR) codes or barcodes, and magnetic response [15–17]. However, these methods have limitations, such as high costs and ease of detection. Over the past few decades, smart luminescent materials have been considered excellent candidates for anti-counterfeiting applications [2, 18–21]. Several luminescent materials, such as semiconductor quantum dots, fluorescent organic dyes, and rare-earth doped materials, have been explored for anti-counterfeiting applications [15]. However, these luminescent materials have limitations; for example, fluorescent organic dyes suffer from low photostability, while semiconductor quantum dots and rare-earth-doped materials are known to be toxic. Recently, all-inorganic perovskite CsPbX_3_ nanocrystals have been predicted as emerging luminescent materials for use in fluorescent labels against counterfeiting due to their excellent optical properties [22–24]. Although a few reports have reported using CsPbX_3_ nanocrystals in anti-counterfeiting, CsPbX_3_ nanocrystals suffer from low photostability, thermal stability, and air/moisture stability [3]. These shortcomings limit their application in anti-counterfeiting. Therefore, various approaches have been employed to improve the stability of CsPbX_3_ nanocrystals [25, 26], and the encapsulation of CsPbX_3_ nanocrystals with water-resistant environmentally inert or stable materials, such as silica, can be an effective approach to improve their stability [3]. Furthermore, paper based on luminescent materials is also becoming as a promising candidate for anti-counterfeiting application. Consequently, significant efforts have been devoted in the development of luminescent paper for anti-counterfeiting applications in recent time which reveals that these paper could be an excellent applicant for anti-counterfeiting application [27–29]. In most of these efforts, luminescent paper based on organic compounds and dyes have many drawbacks such as photostability, low PLQY, high cost, complex and multistep synthesis route, low water-solubility [27, 28, 30–35]. Hence, there is still needed to develop luminescent paper fabricated with luminescent materials which have facile synthesis process, high PLQY, high water stability and biocompatible. Beside this, luminescent paper has another advantage over luminescent inks of nanocrystals is that nanocrystals can be up-taken by cells or tissues due to its nanoscale which suggest that embedding nanocrystals in nanofiber-based paper will improve the bio-compatibility as compared to NCs ink.

Herein, we demonstrate a facile method for fabricating water-stable and highly luminescent polymeric fibers entrapping silica-coated CsPbBr_3_ perovskite nanocrystals (CsPbBr_3_@SiO_2_) using a conventional electrospinning method and present their potential application in anti-counterfeiting and biomedical applications. Biodegradable and biocompatible polycaprolactone (PCL) was used as a polymeric fiber material. The resulting highly luminescent PCL fibers (PCL-perovskite fibers) exhibit a bright green emission at 520 nm, which originates from the CsPbBr_3_@SiO_2_ nanocrystals. The PCL fibers and CsPbBr_3_@SiO_2_ nanocrystals were examined via different techniques, such as SEM, TEM, photoluminescence (PL) and ultra-violet (UV) spectroscopy, fluorescent microscopy, thermogravimetric analysis (TGA), and water contact angle measurements, to analyze their optical, surface, microstructural, and physical properties. Furthermore, the fabricated fluorescent PCL-perovskite fibers were used as paper for printing various patterns to combat counterfeiting. These patterns are only visible when illuminated under UV light (365 nm). In addition, the biocompatibility of the CsPbBr_3_@SiO_2_ nanocrystals and PCL-perovskite fibers was evaluated using a cell viability study. The results indicate that PCL fibers entrapping CsPbBr_3_@SiO_2_ have great potential as luminescent materials for biocompatible anti-counterfeiting applications.

## Experimental and characterization

Chemicals: PbBr_2_ (99.999%), CsBr (99.99%), OAm (70%), OA (90%), TMOS, DMF, PCL and anhydrous toluene (99.95%) were purchased from Sigma–Aldrich. DMEM media (Gibco, USA) fetal bovine serum (FBS) (CellSera, Australia), CCK-8 (Dojindo, Japan), trypsin–EDTA (Gibco, NY), and phalloidin (Invitrogen, Carlsbad, CA, USA) were used as received. These materials were used without additional purification.

### Synthesis of CsPbBr_3_@SiO_2_ core–shell perovskite nanocrystals

CsPbBr_3_@SiO_2_ core–shell perovskite nanocrystals were synthesized according to our previous report [3]. Briefly, CsPbBr_3_@SiO_2_ core–shell perovskite nanocrystals were synthesized from 0.1468 g of PbBr_2_ and 0.0851 g of CsBr dissolved in 10 mL of DMF at 100 °C. Before this, 0.6 mL of OAm and 1.8 mL of OA were added to 10 mL of DMF. When the PbBr_2_ and CsBr were completely dissolved, the solution was cooled to 25 °C. Thereafter, ammonia solution (40 μL, 2.8%) was added to 2 mL of above solution. Toluene (20 mL) containing 10 μL of TMOS was stirred at 1500 rpm, while 0.4 mL of the precursor solution was quickly injected into the toluene solution under stirring at 1500 rpm. When the above precursor solution was injected into the toluene solution, it became yellow. CsPbBr_3_ perovskite nanocrystals were formed immediately after injection due to their poor solubility. After 20–25 s, the stirring speed was adjusted to 150 rpm and maintained for 24 h. The final sample was collected via centrifugation at 10,000 rpm for 10 min.

### Fabrication of PCL-perovskite electrospun fibers and films

PCL and PCL-perovskite electrospun fibers were prepared by a conventional electrospinning process. Briefly, PCL (1 g) was dissolved in 2,2,2-trifluoroethanol to obtain a 20 wt% solution and 2% (w/w) of CsPbBr_3_@SiO_2_ core–shell perovskite nanocrystals (20 mg) was mixed with this solution. The PCL solution was loaded into a syringe, electrospun for 1 h, and ejected through a 23G needle at a voltage of 8.0 kV and flow rate of 1 mL/h. For the aligned fibers, the collecting wafers were taped onto a rotating stainless-steel drum spun at 1000 rpm. The electrospun meshes were collected on aluminum foil.

### Characterization

SEM images were obtained on a SEM (JSM‐7001F, JEOL Ltd., Tokyo, Japan), and the water contact angles were determined via water contact angle analyzer (Phoenix‐MT, Surface Electro Optics, Suwon, Korea). TEM images were obtained on a transmission electron microscope (Philips Electron Optics, Netherlands) at an acceleration voltage of 15 kV. The thermal stability of the fiber film was evaluated using a Mettler Toledo 851e thermal system (Zurich, Switzerland) at a heating rate of 10 °C/min under a nitrogen atmosphere (flow rate: 10 mL/min). The PL spectra of the samples were recorded on a Perkin LM555 spectrometer (PerkinElmer, Waltham, Massachusetts, United States).

### Cell culture

Adipose derived stem cell (ADSC) were cultured in Dulbecco's modified Eagle's medium–low glucose (DMEM L/G) supplemented with 10% (v/v) FBS and 1% (v/v) penicillin/ streptomycin (P/S) under a humidified atmosphere comprised of 95% air and 5% CO_2_ at 37 °C.

### Cell proliferation assay

The biocompatibility of the PCL-perovskite fiber paper was determined by studying its cell proliferation ability using a cell counting kit (CCK-8). Before cell seeding, the fibers were sterilized with ethanol and UV light and then placed in a 24-well culture plate. Subsequently, the ADSC suspension (2 × 10^4^ cells/well) was seeded onto the culture plate. After 1, 3, and 7 d of cell culture, the growth medium was replaced with CCK solution (10% v/v in medium) and incubated for 3 h. Thereafter, the absorbance was measured at 450 nm using a microplate reader (Versa Amax, USA). Cells grown on the tissue culture dishes served as the control.

### Cell skeleton staining with phalloidin

Random and aligned fiber meshes were sterilized with ethanol and UV before cell seeding. The cells were seeded into the fiber mesh at a cell density of 4 × 10^4^ cells/well and incubated for 24 h. After the time of cell culture, the cell-seeded fibers were permeabilized with 0.1% Triton- × 100 followed by paraformaldehyde (4%) fixation. Thereafter, the cells were incubated with phalloidin solution as per manufacturer's instructions for 1 h at room temperature. DAPI (Molecular Probes, Eugene, OR, USA) was used to stain the nuclei and visualized under a confocal microscope (Zeiss LSM700, Germany).

## Results and discussion

Water-stable, non-toxic and highly luminescent CsPbBr_3_@SiO_2_ nanocrystals are prepared via a simple chemical method. The mechanism for the growth of the SiO_2_ coating around CsPbBr_3_ has been explained in detail in our previous study [3]. Briefly, the injection of silica precursor solution made tetramethylorthosilicate (TMOS) oligomers start to adsorb on the perovskite nanocrystals (PNCs) surface of CsPbBr_3_ after, which results in the formation of the CsPbBr_3_@SiO_2_ nanocrystals. The crystal structure of CsPbBr_3_@SiO_2_ nanocrystals was investigated using Powder X-ray diffraction (XRD) (Additional file 1: Figure S1). The XRD pattern of CsPbBr_3_@SiO_2_ nanocrystals exhibit diffraction peaks at 15.1°, 12.36°, 24.08°, 26.28°, 30.2°, 34.33°, 37.6°, 43.52°, 46.32°, 49.28°, which correspond to (001), (110), (− 1, 1, 0), (111), (002), (− 201), (121), (− 202), (− 212) and (− 301) planes of CsPbBr_3_. The XRD pattern of CsPbBr_3_@SiO_2_ nanocrystals was in good agreement with JCPDS:00-018-0364, which reveals that the crystal structure of CsPbBr_3_@SiO_2_ nanocrystals is monoclinic and remain unchanged and not affected by the SiO_2_ encapsulation process [36, 37].

Furthermore, X-ray photoelectron spectroscopy (XPS) was carried out for the analysis of surface elemental composition. The XPS results obtained for CsPbBr_3_@SiO_2_ nanocrystals are shown in Additional file 1: Figure S2. The survey scanned XPS spectrum (Additional file 1: Figure S2(a)) shows various peaks related to Pb^5d^, Br^3d^, Cs^4d^, Si^2p^, Pb^4f^, Cs^4p^, Br^3p^, O^1s^, and Cs^3d^ at binding energies of 24, 68, 76, 103, 143, 155, 181, 533, and 724 eV, respectively [38]. Moreover, the characteristics of chemical bonds between the various elements, such as Cs, Pb, Br, Si, and O, were also analyzed. The XPS spectra obtained Cs^3d^, Pb^4f^, Br^3d^, Si^2p^, and O^1s^ are shown in Additional file 1: Figure S2(b–f). Additional file 1: Figure S2(b) shows the Cs^3d^ XPS spectrum, which exhibits two peaks at binding energies of 724 and 738 eV attributed to inner and surface ions, respectively [39–41]. Additional file 1: Figure S2(c) shows the XPS spectrum of Pb^4f^, which shows the peaks at binding energies of 138.5 and 143.5 eV corresponding to the (4f_7/2_) and Pb (4f_5/2_) core energy levels of the Pb^2+^ cations, respectively [39, 42]. The two additional peaks before 138.5 and 143.5 eV maybe associated with metallic Pb^0^ (4f_7/2_ and Pb 4f_5/2_), respectively. Additional file 1: Figure S2(d) shows the XPS spectrum obtained for Br^3d^, which reveals two Br 3d peaks corresponding to 3d_3/2_ and 3d_5/2_ peaks. The XPS spectra obtained for Si 2p, and O 1 s exhibit peaks at 103.5, and 532.7 eV, respectively, as shown in Additional file 1: Figure S2(e–f) which attributed to formation SiO_2_ shell [23, 43].

**Fig. 1:**
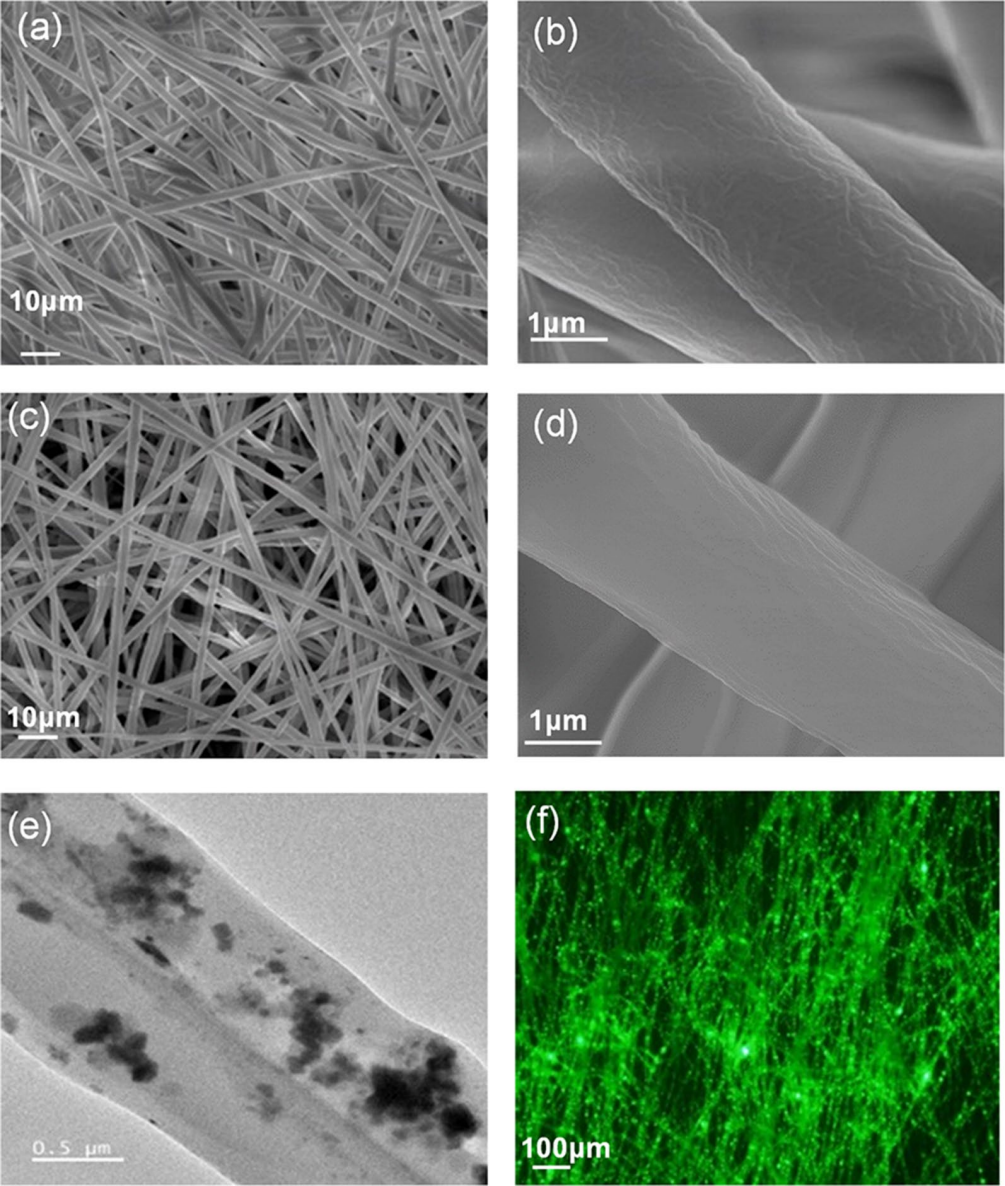
Images of PCL and PCL-perovskite fibers. **a** Low and **b** High-resolution SEM image of the PCL fibers, **c** Low and **d** High-resolution SEM image of the PCL-perovskite fibers. **e** TEM and **f** Fluorescent images of the PCL-perovskite fibers

The SEM and TEM were performed to examine the surface morphology and microstructure of the fabricated PCL and PCL-perovskite fibers. The SEM images of PCL and the PCL-perovskite fibers are shown in Fig. 1a–d. Figure 1a and c show that PCL and PCL-perovskite-based fibers have a highly dense net-like distribution, which was uniform throughout. High-resolution SEM images of the fibers were recorded to examine the surface of the individual fibers. The high-resolution SEM images show that the PCL and PCL-perovskite-based fibers have smooth surfaces (Fig. 1b and d). The observed diameters of PCL and the PCL-perovskite fibers were 2.4 ± 0.46 and 2.5 ± 0.15 μm, respectively. These results reveal that CsPbBr_3_@SiO_2_ nanocrystals were perfectly entrapped within the fibers and did not affect the electrospinning process. Therefore, incorporated CsPbBr_3_@SiO_2_ nanocrystals did not affect the fiber morphology and diameter. These conclusions were further confirmed by the TEM analysis. The TEM image of the PCL-perovskite fibers is shown in Fig. 1e, which confirmed that CsPbBr_3_@SiO_2_ nanocrystals were embedded inside the fibers. Furthermore, the fluorescent image of the PCL-perovskite fibers under UV light is shown in Fig. 1f, which exhibits a bright green emission throughout the fibers, revealing that CsPbBr_3_@SiO_2_ nanocrystals were uniformly distributed in the PCL-perovskite fibers. Moreover, aligned PCL-perovskite fibers were fabricated using PCL and CsPbBr_3_@SiO_2_ nanocrystals. The fibers were aligned and oriented during the electrospinning process. Control over the fiber alignment is highly desirable for producing anisotropic meshes with increased complexity and performance. In addition, aligned fibers can improve the mechanical properties because crystallinity is increased during electrospinning process [44]. The SEM and fluorescent images of the aligned PCL-perovskite fibers are shown in Additional file 1: Figure S3, indicating that the aligned PCL-perovskite fibers were uniformly fabricated and highly luminescent under UV light. The uniform fluorescence form PCL-perovskite fibers and aligned PCL-perovskite fibers as illustrated in fluorescent images in Fig. 1f and Additional file 1: Figure S3(b) clearly demonstrated that CsPbBr_3_@SiO_2_ nanocrystals are uniformly distributed in PCL-perovskite fibers.

**Fig. 2:**
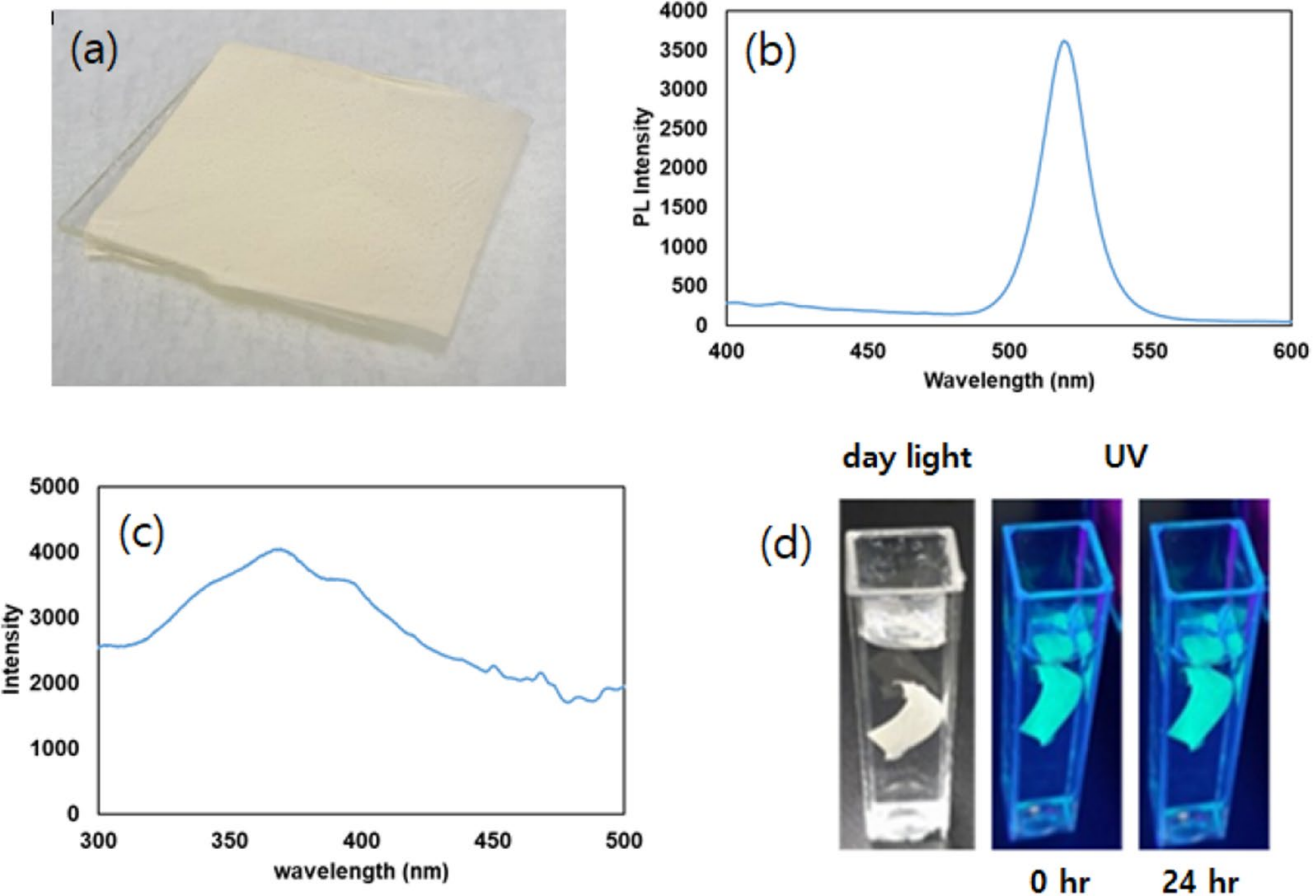
Optical properties of the PCL-perovskite fibers. **a** Photograph of the fabricated PCL-perovskite fibers. **b** PL emission spectrum obtained for the PCL-perovskite fibers. **c** PL excitation spectrum obtained for the PCL-perovskite fibers. **d** Photograph of the PCL-perovskite fibers in water expose to normal light and UV light

The optical features of the as-synthesized CsPbBr_3_@SiO_2_ nanocrystals and PCL-perovskite fibers were evaluated using ultraviolet–visible (UV–Vis) absorption and PL. Additional file 1: Figure S4 shows the UV–Vis absorption spectrum obtained for the as-synthesized CsPbBr_3_@SiO_2_ nanocrystals, which exhibit a broad absorption peak centered at 492 nm, and was consistent with those previously reported [45, 46]. The PL emission spectra obtained for water dispersed as-synthesized CsPbBr_3_@SiO_2_ nanocrystals and after storage in water for 24 h are displayed in Additional file 1: Figure S5. The observed the PLQY of CsPbBr_3_@SiO_2_ was ~ 10.2. The PL emission spectrum of CsPbBr_3_@SiO_2_ nanocrystals exhibit a bright green emission at 520 nm upon excitation at 374 nm (Additional file 1: Figure S5). In addition, the emission intensity of CsPbBr_3_@SiO_2_ nanocrystals did not decrease significantly with ~ 80% of its initial intensity being retained after prolonged storage in water for 24 h, indicating that the as-synthesized CsPbBr_3_@SiO_2_ nanocrystals were water-stable (Additional file 1: Figure S5). A photograph of the fabricated PCL-perovskite fiber paper is presented in Fig. 2a. The PL emission spectrum obtained for the PCL-perovskite-based fibers is shown in Fig. 2b, which reveals that the PL emission of the PCL-perovskite fibers was the same as that of the CsPbBr_3_@SiO_2_ nanocrystals and centered at 520 nm upon excitation at 374 nm. The PL excitation spectrum of PCL-perovskite fibers is shown in Fig. 2c. The photoluminescence excitation (PLE) spectrum shows that the PCL-perovskite fibers have a broad excitation spectrum with a maximum observed at 374 nm. The PCL-perovskite fibers were immersed in water to evaluate their water stability. Figure 2d shows the PCL-perovskite fibers in water under normal light and UV light. The PCL-perovskite fibers exhibit a bright emission even after storage in water for 24 h. Moreover, the comparison between the water stability of PCL-perovskite fibers fabricated using CsPbBr_3_ and CsPbBr_3_@SiO_2_ nanocrystals is illustrated in Additional file 1: Figure S6 which clearly demonstrate that green emission form PCL-perovskite fiber made-up with CsPbBr_3_@SiO_2_ nanocrystals still same while decreases in case of PCL-perovskite fiber made-up with CsPbBr_3_ nanocrystals which is be due to the silica shell in CsPbBr_3_@SiO_2_ nanocrystals.

Hydrophobicity is important to develop stable and efficient anti-counterfeiting product. Therefore, the water contact angles were measured to examine the hydrophobicity of PCL and the PCL-perovskite fibers. The contact angle images of PCL and the PCL-perovskite-based fibers are shown in Additional file 1: Figure S7 and were determined to be 66° and 68°, respectively. The results show that there were no changes in the water contact angle upon the addition of the perovskite nanocrystals. TGA of the PCL and PCL-perovskite fibers were performed to evaluate their thermal properties. Figure S8 shows that the degradation of both samples occurred between 300 and 475 °C. Early weight loss was due to the loss of water. The remaining weight loss was due to the loss of the organic component (PCL). The results show that adding perovskite provides thermal stability similar to that of the PCL fibers. As the TGA was conducted under an N_2_ atmosphere, the remaining weight of the PCL fiber can be attributed to the charred organic residue. The difference in weight between PCL (1.127%) and PCL-perovskite fibers (1.813%) can be attributed to presence of the CsPbBr_3_@SiO_2_ nanocrystals within PCL-perovskite fibers. As discussed above, contact angle and TGA show that PCL-perovskite fibers have thermal and water stability. Additionally, there are pervious reports which shown that CsPbBr_3_@SiO_2_ nanocrystals have higher photostability and stability [23, 47].

**Fig. 3:**
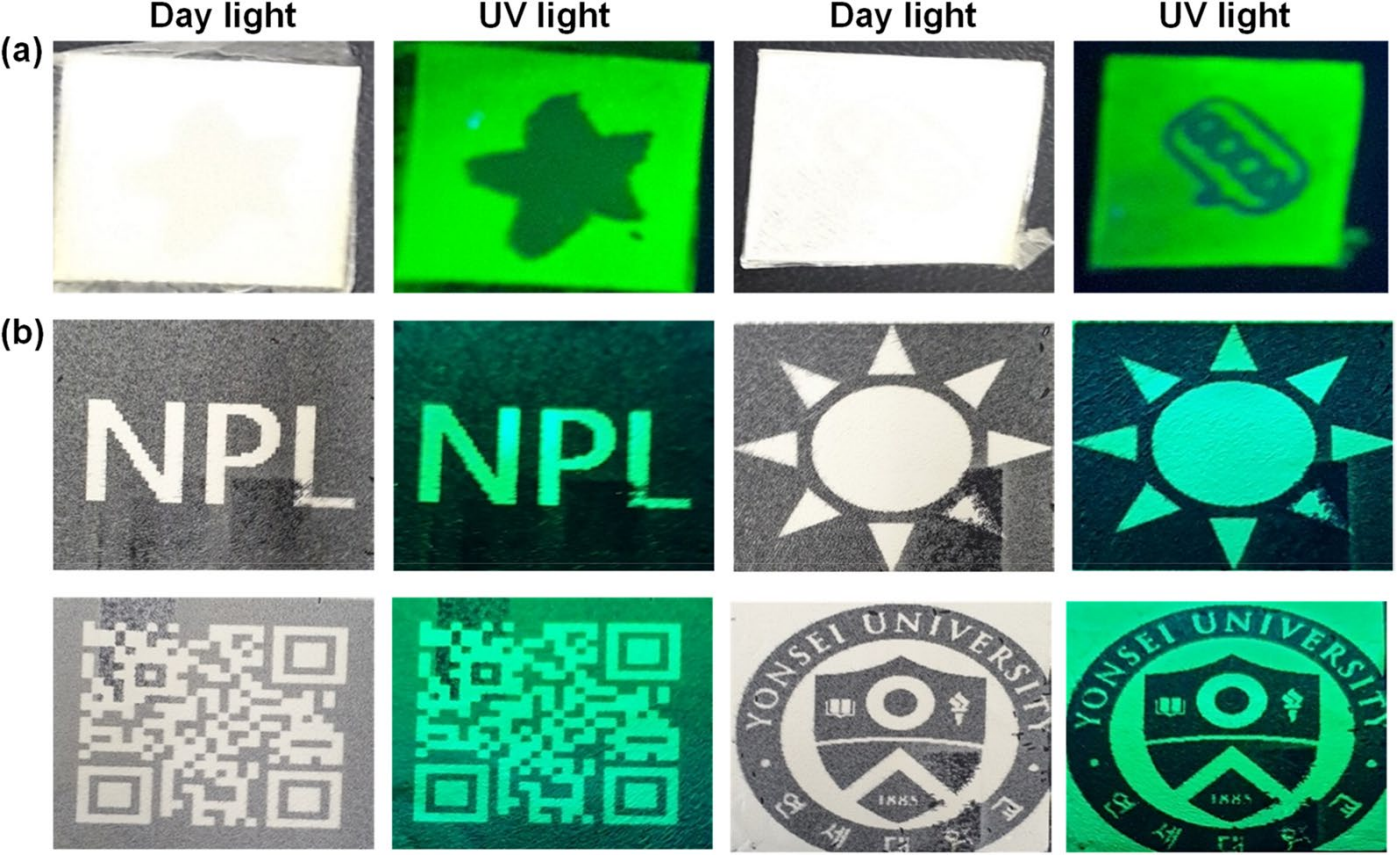
Anti-counterfeiting applications. **a** The pattern stamped on PCL-perovskite fibers-based white paper using white ink under daylight and UV light (365 nm). **b** Alphabet “NPL”, Sun, QR code, and logo of “Yonsei University” patterns printed with black color ink using an inkjet printer via a reverse printing approach on the fluorescent PCL-perovskite fibers-based white paper

**Fig. 4:**
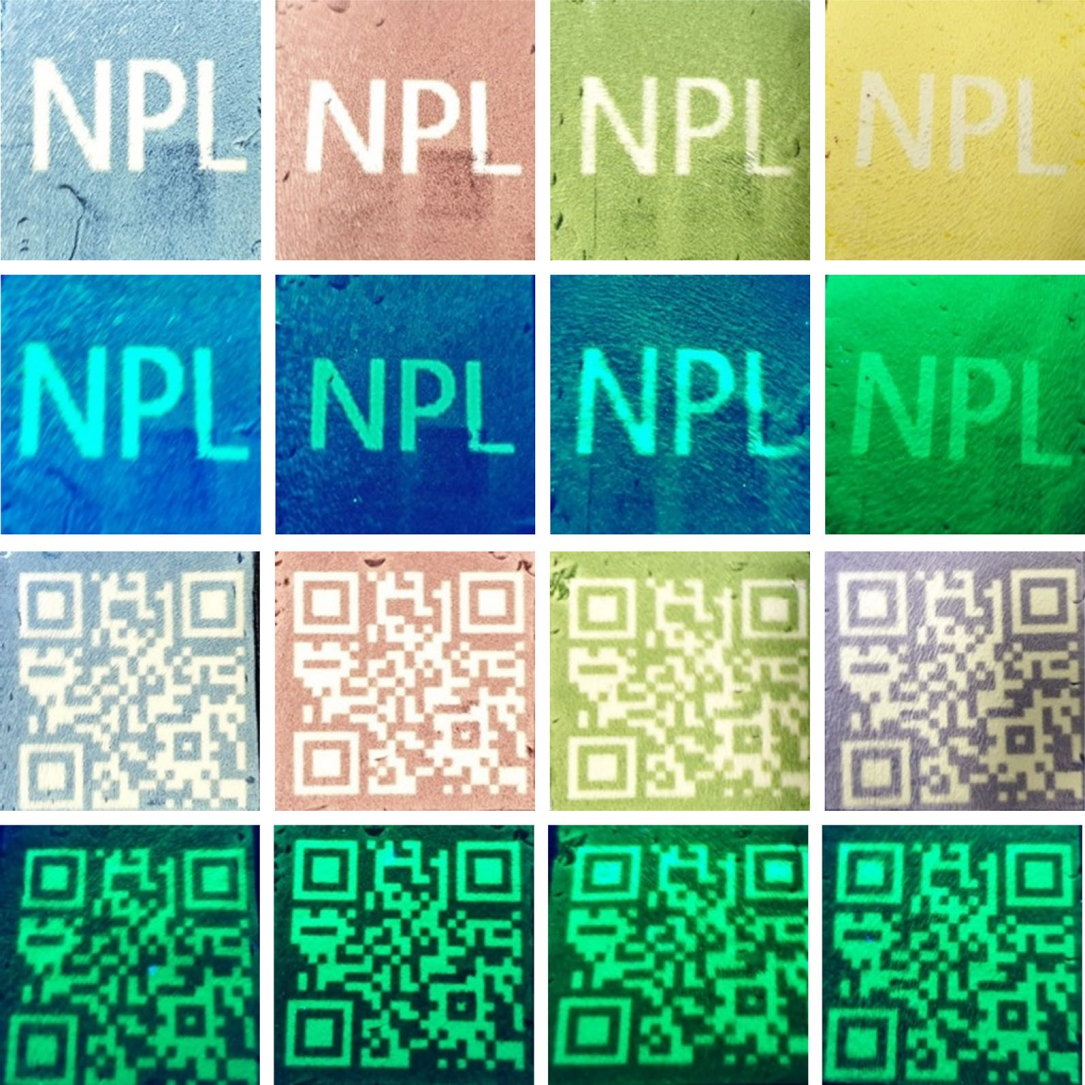
Anti-counterfeiting application. Alphabet “NPL” and QR code patterns printed with blue, brown, green, and yellow on an inkjet printer using a reverse printing approach on fluorescent PCL-perovskite fibers-based white paper

Recently, various luminescent materials, including perovskite nanocrystals have been used in anti-counterfeiting applications. Traditionally, luminescent inks have been used to design invisible security codes to protect against counterfeiting. However, developing luminescent paper is also an efficient method to protect against counterfeiting. In this study, we demonstrate the use of PCL-perovskite fiber paper prepared via an electrospinning method for anti-counterfeiting applications, which has not been reported to date. We have investigated the use of fluorescent PCL-perovskite-based fiber white paper (fluorescent paper) in anti-counterfeiting applications. Invisible patterns were stamped on PCL-perovskite-based fiber white paper using commercially available white ink. Figure 3a shows the pattern stamped on the PCL-perovskite-based fiber paper using white ink under daylight and UV light (365 nm). Figure 3a clearly shows that the stamped pattern was invisible under daylight but can be seen when exposed to UV light at 365 nm. The stamped pattern covers the fluorescent paper and shows no green emission in the stamped area, while the white paper shows a bright green color. Similarly, we printed various patterns, such as an alphabet “NPL,” Sun, QR code, and logo of “Yonsei University”, with black color ink using an inkjet printer via a reverse printing approach on fluorescent PCL-perovskite based fibers white paper (Fig. 3b). In the reverse-printing approach, the paper was covered with printing ink, except for the design pattern. Figure 3b shows that the printed alphabet “NPL”, Sun, QR code, and logo of “Yonsei University” did not show any color in daylight but exhibited a bright green emission when exposed to UV light at 365 nm, which can be seen by the naked eye. Furthermore, we also printed the alphabet “NPL” and QR code with blue, brown, green, and yellow ink on fluorescent PCL-perovskite-based fiber white paper to check the feasibility of ink in different colored backgrounds. The alphabet “NPL” and QR code patterns printed with blue, brown, green, and yellow ink are illustrated in Fig. 4, which shows that the printed patterns exhibit no emission in daylight and a green emission under UV light (365 nm). Furthermore, for prospective practical applications, stability and durability of printed pattern is highly importance [2, 23, 47]. Therefore, the lifetime stability of printed pattern is evaluate in order to see their ability for commercial use. For the time-dependent stability of fluorescent PCL-perovskite fiber paper, a comparative analysis was conducted between a newly generated QR code and one that is over 6 months old. The QR code of two different samples in daylight and UV light is shown in Additional file 1: Figure S9. Additional file 1: Figure S9 demonstrated that QR code exhibits green emission when it exposes to UV light (365 nm) even after 6 months of storage under ambient conditions which proves that printed QR code on these PCL-perovskite fiber paper shows remarkable stability even after 6 months. Additionally, thermal stability of these 6 month-old printed QR code is also examine with heating and cooling cycle at 60 °C and 80 °C. In each heating and cooling cycle, QR code patterns is heated for 10 min at 60 °C or 80 °C and them cooled at room temperature. Additional file 1: Figure S11(a) and (b) reveals that printed QR code are stable in heating and cooling cycle and there in almost no significant change in green emission after 6 cycles of heating and cooling at 60 °C and 80 °C. Consequently, our results revealed that fluorescent PCL-perovskite-based fiber white paper is a promising candidate for anti-counterfeiting applications, where the certification of the original paper can be confirmed upon exposure to UV light at 365 nm.

**Fig. 5:**
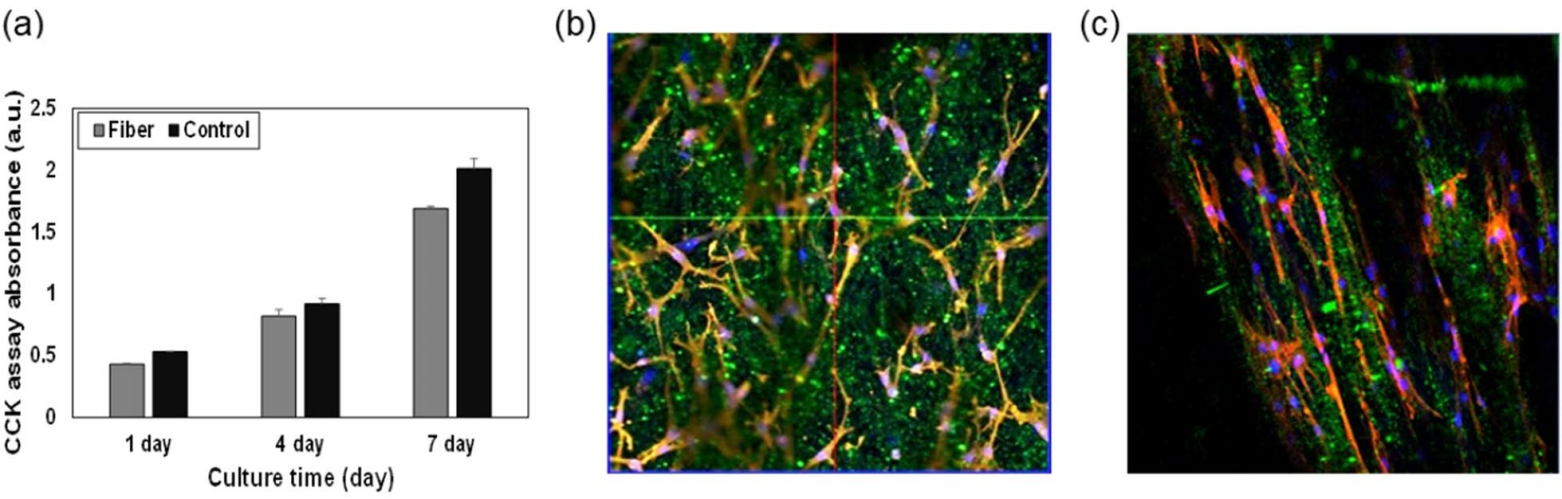
Cytocompatibility of PCL-perovskite fibers. **a** Cell proliferation of ADSC on the PCL-perovskite fibers. Fluorescence microscopy images of DAPI and phalloidin staining of the ADSC on the **b** random and **c** aligned PCL-perovskite-based fibers. DAPI and phalloidin staining of the cell nuclei and actin in ADSC are shown in blue and red, respectively

Biocompatibility is an important parameter that determines the commercial use of materials. The anti-counterfeiting is promising technique used to combat against forging of high-value documents, medicines and pharmaceuticals packing, banknotes, daily goods etc. In most of above discussed anti-counterfeiting applications involves human contact. Therefore, biocompatibility and non-toxic are important of anti-counterfeiting application which is also well established in previous studies [48–52]. Hence, we preformed biocompatibility studies in present study. The biocompatibility of the PCL-perovskite fibers was determined using a CCK assay utilizing ADSC. The results show that the proliferation of ADSC on the fibers was increased, similar to that of the control (Fig. 5a). Therefore, the CCK assay results indicate that the PCL-perovskite fiber paper was not cytotoxic. In addition, cell attachment on the PCL-perovskite fiber paper was confirmed upon phalloidin staining on both the random and aligned PCL-perovskite-based fiber scaffolds (Fig. 5b and c). The cell attachment and spreading of the ADSC led to good biocompatibility in vitro [53]. Moreover, CsPbBr_3_@SiO_2_ nanocrystals have been for explored for bio-medical application due to their biocompatibility [3, 43, 54]. Therefore, owing to biocompatibility CsPbBr_3_@SiO_2_ nanocrystals and PCL-perovskite fiber can also be utilizing for various biomedical applications.

Based on these results, it is expected that fluorescent and biocompatible PCL-perovskite-based fiber paper will be used in biocompatible anti-counterfeiting applications. Using PCL-perovskite-based fiber white paper for anti-counterfeiting applications may offer an opportunity to design security features for authorizing certificates or secret documents with high-end security against counterfeiting and encryption.

## Conclusions

We have successfully prepared bright green PCL-perovskite fiber paper using a simple electrospinning process. The fabricated PCL-perovskite fibers exhibit a bright green emission at 520 nm. The PL emission in water, TGA, and contact angle measurements reveal that the PCL-perovskite fibers have high thermal and water stability. Various patterns with multiple colors have been generated on the PCL-perovskite fibers, which demonstrated the potential of the proposed PCL-perovskite fiber paper for anti-counterfeiting. Cytotoxicity tests showed that the PCL-perovskite fibers were biocompatible, making them suitable for biomedical anti-counterfeiting applications. Fluorescent PCL-perovskite fiber paper would be suitable for designing security signs, letters, patterns, and confidential documents against counterfeiting. The obtained results indicate that this study may open a new avenue for emerging advanced anti-counterfeiting and encryption applications.

## Supplementary Information

**Additional file 1: Fig. S1.** The XRD pattern of CsPbBr_3_@SiO_2_ nanocrystals. **Fig. S2.** XPS analysis of CsPbBr_3_@SiO_2_ nanocrystals; (a) survey scan and (b-f) X-ray photoelectron spectra of Cs^3d^, Pb^4f^, Br^3d^, Si^2p^, and O^1s^, respectively. **Fig. S3.** (a) and (b) The SEM and fluorescent images of aligned PCL-perovskite fiber, respectively. **Fig. S4.** The UV–Vis absorption spectrum of the CsPbBr_3_@SiO_2_ nanocrystals. **Fig. S5.** PL emission spectrum of CsPbBr_3_@SiO_2_ nanocrystals in water and after 24 h. **Fig. S6.** Photographs of the PCL-perovskite fibers fabricated with (a) CsPbBr_3_ and (b) CsPbBr_3_@SiO_2_ nanocrystals in water under normal daylight and UV light at initial time and after 24 h. **Fig. S7.** Water contact angle of (a) PCL, and (b) PCL-Perovskite fiber surfaces. **Fig. S8.** The thermogravimetric analyses of PCL and PCL-Perovskite fiber. **Fig. S9.** Photographs of newly printed QR code and after 6 months at ambient condition on PCL-perovskite fiber-based white paper using brown ink under daylight and UV light (365 nm). **Fig. S10.** Photographs of the QR code patterns printed on PCL-perovskite fibers-based paper under daylight and UV light (365 nm) at (a) 60 °C and (b) 80 °C at different time interval. **Fig. S11.** Thermal stability heating cycles: photographs of the QR code printed on PCL-perovskite fibers-based paper, taken before and after heating at different cycle under UV light (365 nm) at (a) 60 °C and (b) 80 °C.

## Data Availability

The data that support the findings of this study are available from the authors on reasonable request.
