# Supplementary material for: Water-stable, biocompatible, and highly luminescent perovskite nanocrystals-embedded fiber-based paper for anti-counterfeiting applications
Source: Nano Converg. 2023 May 3;10:21. doi: 10.1186/s40580-023-00366-6 (PMC10156878; doi:10.1186/s40580-023-00366-6)
Supplement: Supplementary file 1 — Additional file 1: Fig. S1. The XRD pattern of CsPbBr3@SiO2 nanocrystals. Fig. S2. XPS analysis of CsPbBr3@SiO2 nanocrystals; (a) survey scan and (b-f) X-ray photoelectron spectra of Cs3d, Pb4f, Br3d, Si2p, and O1s, respectively. Fig. S3. (a) and (b) The SEM and fluorescent images of aligned PCL-perovskite fiber, respectively. Fig. S4. The UV–Vis absorption spectrum of the CsPbBr3@SiO2 nanocrystals. Fig. S5. PL emission spectrum of CsPbBr3@SiO2 nanocrystals in water and after 24 h. Fig. S6. Photographs of the PCL-perovskite fibers fabricated with (a) CsPbBr3 and (b) CsPbBr3@SiO2 nanocrystals in water under normal daylight and UV light at initial time and after 24 h. Fig. S7. Water contact angle of (a) PCL, and (b) PCL-Perovskite fiber surfaces. Fig. S8. The thermogravimetric analyses of PCL and PCL-Perovskite fiber. Fig. S9. Photographs of newly printed QR code and after 6 months at ambient condition on PCL-perovskite fiber-based white paper using brown ink under daylight and UV light (365 nm). Fig. S10. Photographs of the QR code patterns printed on PCL-perovskite fibers-based paper under daylight and UV light (365 nm) at (a) 60 °C and (b) 80 °C at different time interval. Fig. S11. Thermal stability heating cycles: photographs of the QR code printed on PCL-perovskite fibers-based paper, taken before and after heating at different cycle under UV light (365 nm) at (a) 60 °C and (b) 80 °C. [file 40580_2023_366_MOESM1_ESM.docx]

Supplementary Information

**Water-stable, biocompatible, and highly luminescent perovskite nanocrystals-embedded fiber-based paper for anti-counterfeiting applications**

Madhumita Patel,^a^ Rajkumar Patel,^b^ Chanho Park,^c^ Kanghee Cho,^a^ Pawan Kumar,^c,^ * Cheolmin Park ^c^ and Won-Gun Koh,^a,^ *

^a^Department of Chemical and Biomolecular Engineering, Yonsei University, 50 Yonsei-ro, Seodaemun-gu, Seoul 120-749, South Korea.

^b^Energy & Environmental Science and Engineering (EESE), Integrated Science and Engineering Division (ISED), Underwood International College, Yonsei University, 85 Songdogwahak-ro, Yeonsu-gu, Incheon 21983, South Korea

^c^Department of Materials Science and Engineering, Yonsei University, 50 Yonsei-ro, Seodaemun-gu, Seoul 120-749, South Korea.

^∗^*Corresponding author*: Pawan Kumar, Won-Gun Koh and Cheolmin Park

*Email addresses:* [*pawankumar.nst@gmail.com*](mailto:pawankumar.nst@gmail.com) *(P. Kumar),* [cmpark@yonsei.ac.kr](mailto:cmpark@yonsei.ac.kr) (C. Park), and [*wongun@yonsei.ac.kr*](mailto:wongun@yonsei.ac.kr) *(*W-G Koh)


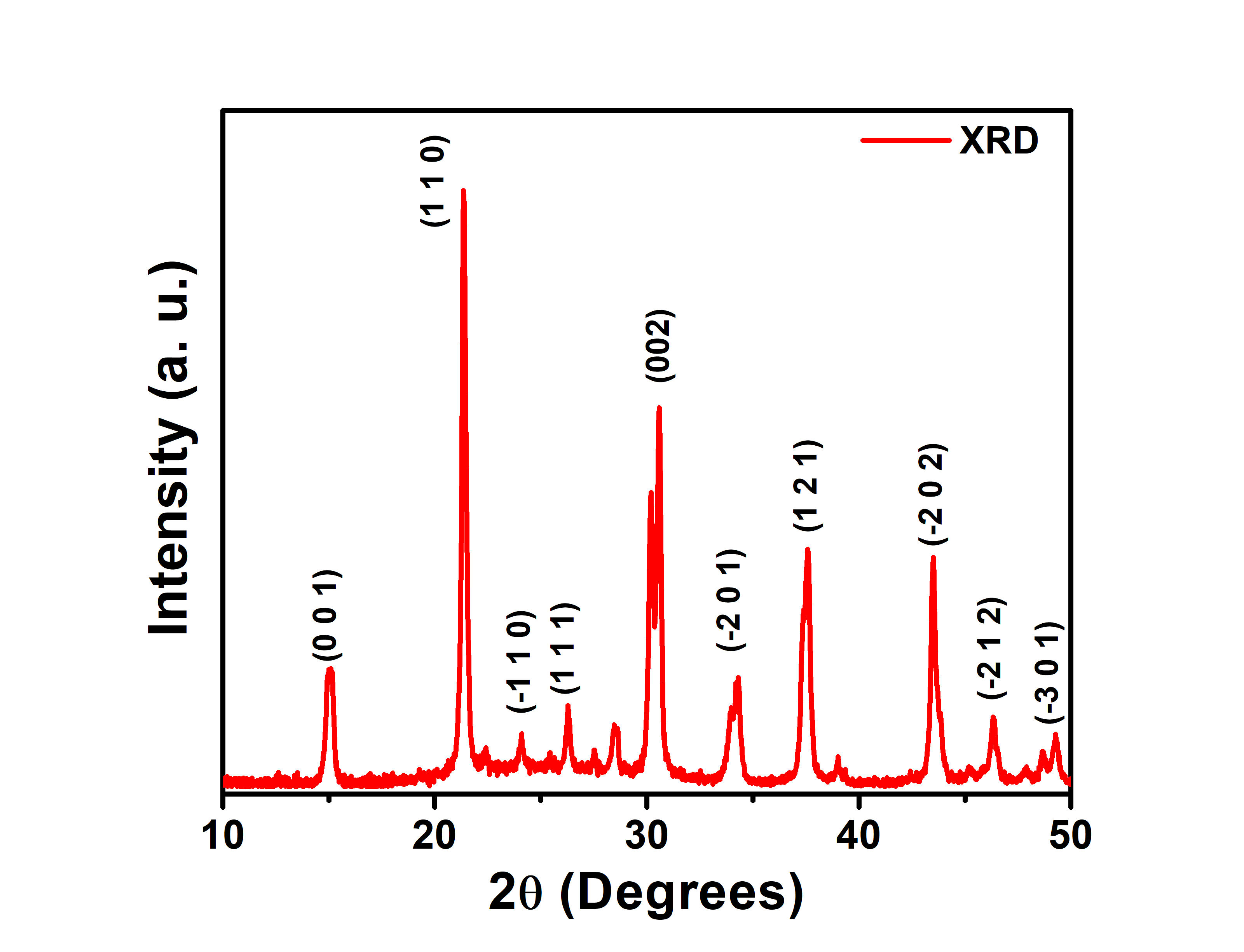


**Fig. S1:** The XRD pattern of CsPbBr_3_@SiO_2_ nanocrystals.


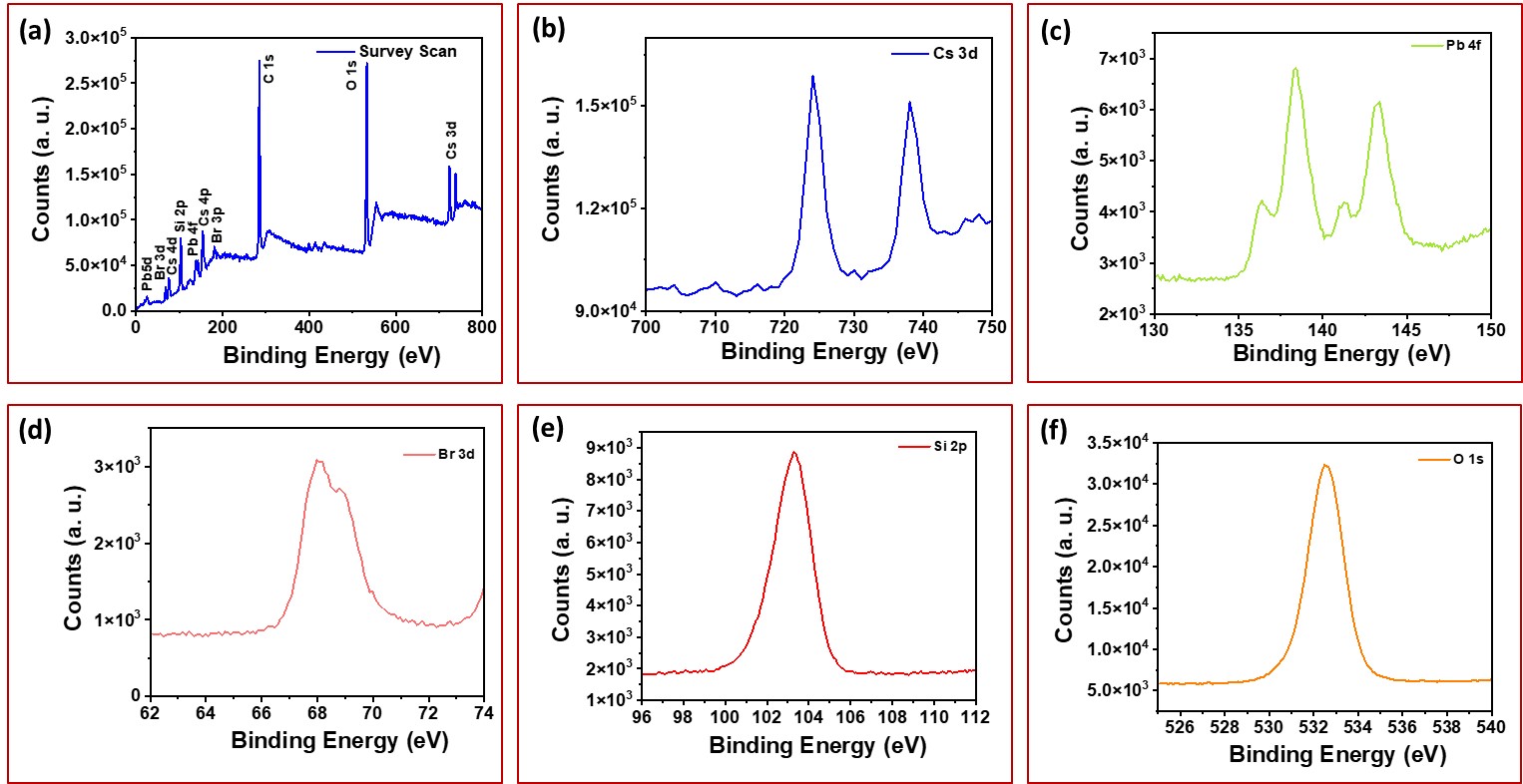


**Fig. S2:** XPS analysis of CsPbBr_3_@SiO_2_ nanocrystals; (a) survey scan and (b-f) X-ray photoelectron spectra of Cs^3d^, Pb^4f^, Br^3d^, Si^2p^, and O^1s^, respectively.


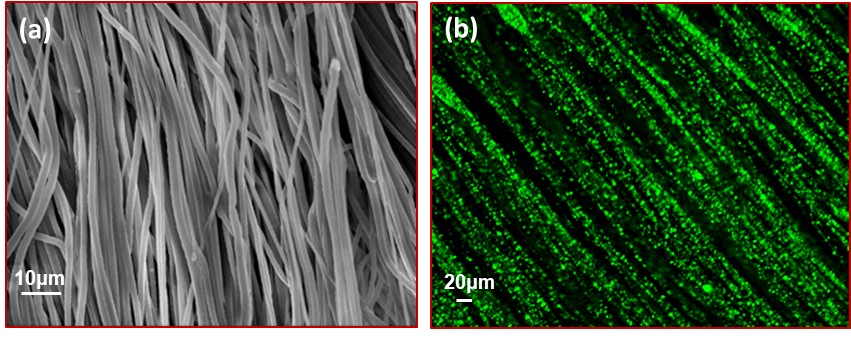


**Fig. S3:** (a) and (b) The SEM and fluorescent images of aligned PCL-perovskite fiber, respectively.


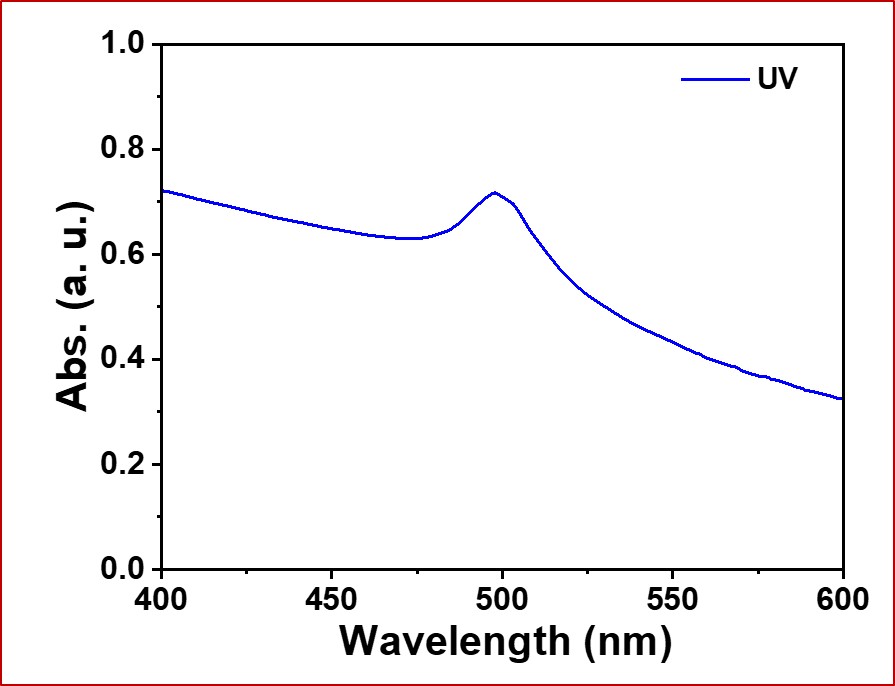


**Fig. S4:** The UV-Vis absorption spectrum of the CsPbBr_3_@SiO_2_ nanocrystals


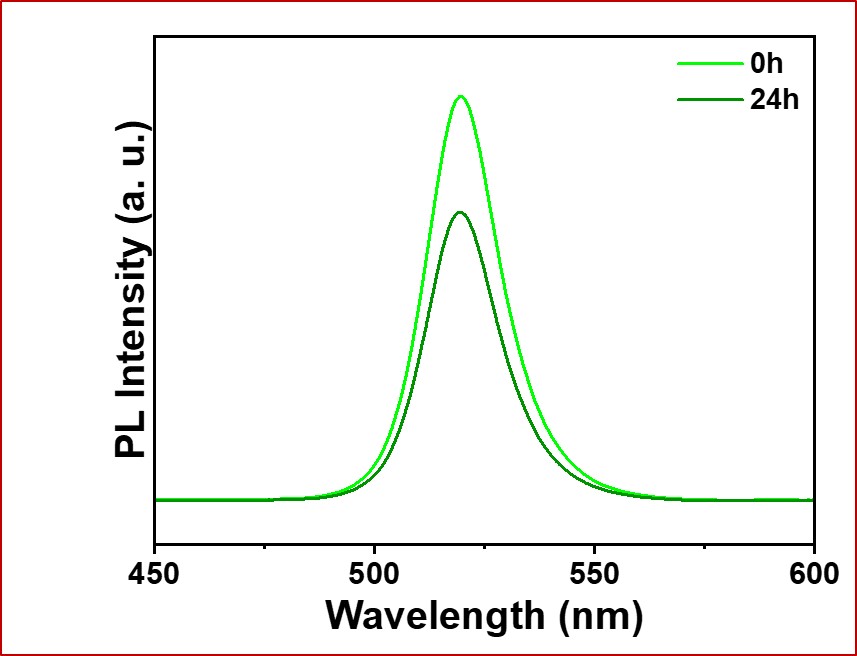


**Fig. S5:** PL emission spectrum of CsPbBr_3_@SiO_2_ nanocrystals in water and after 24 hrs.


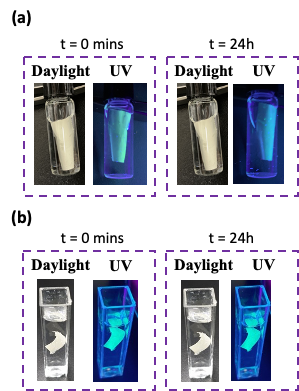


**Fig. S6:** Photographs of the PCL-perovskite fibers fabricated with (a) CsPbBr_3_ and (b) CsPbBr_3_@SiO_2_ nanocrystals in water under normal daylight and UV light at initial time and after 24 hours.


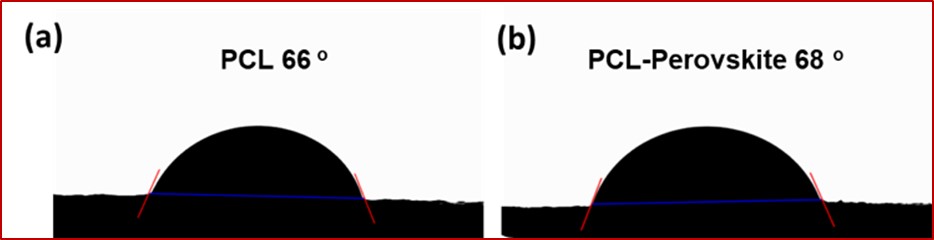


**Fig. S7**: Water contact angle of (a) PCL, and (b) PCL-Perovskite fiber surfaces.


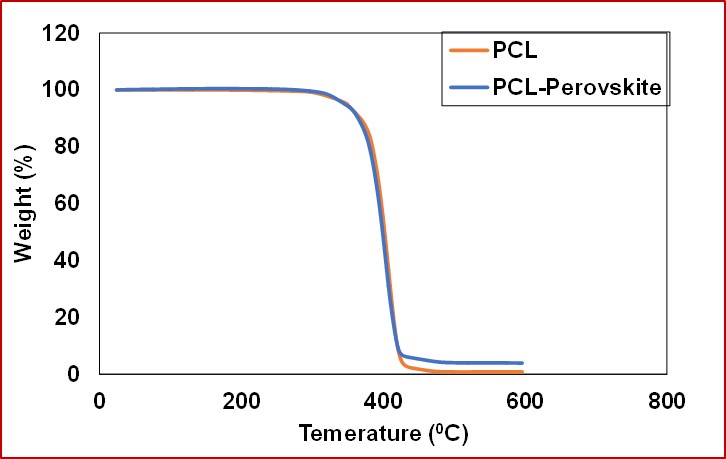


**Fig. S8:** The thermogravimetric analyses of PCL and PCL-Perovskite fiber


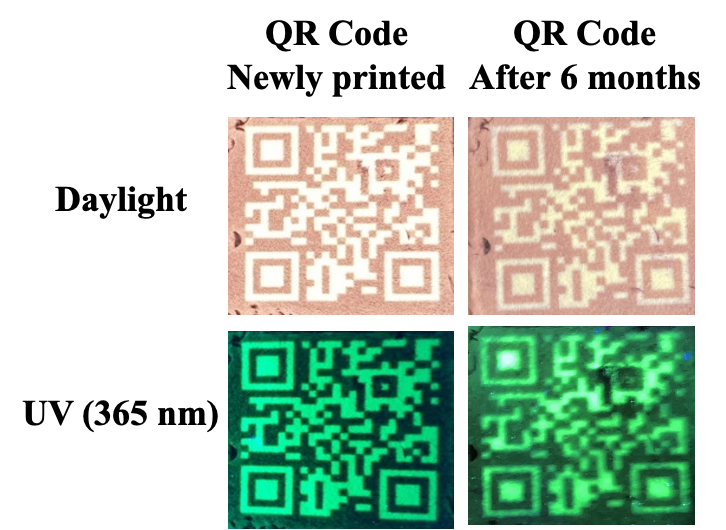


**Fig. S9:** Photographs of newly printed QR code and after 6 months at ambient condition on PCL-perovskite fiber-based white paper using brown ink under daylight and UV light (365 nm).


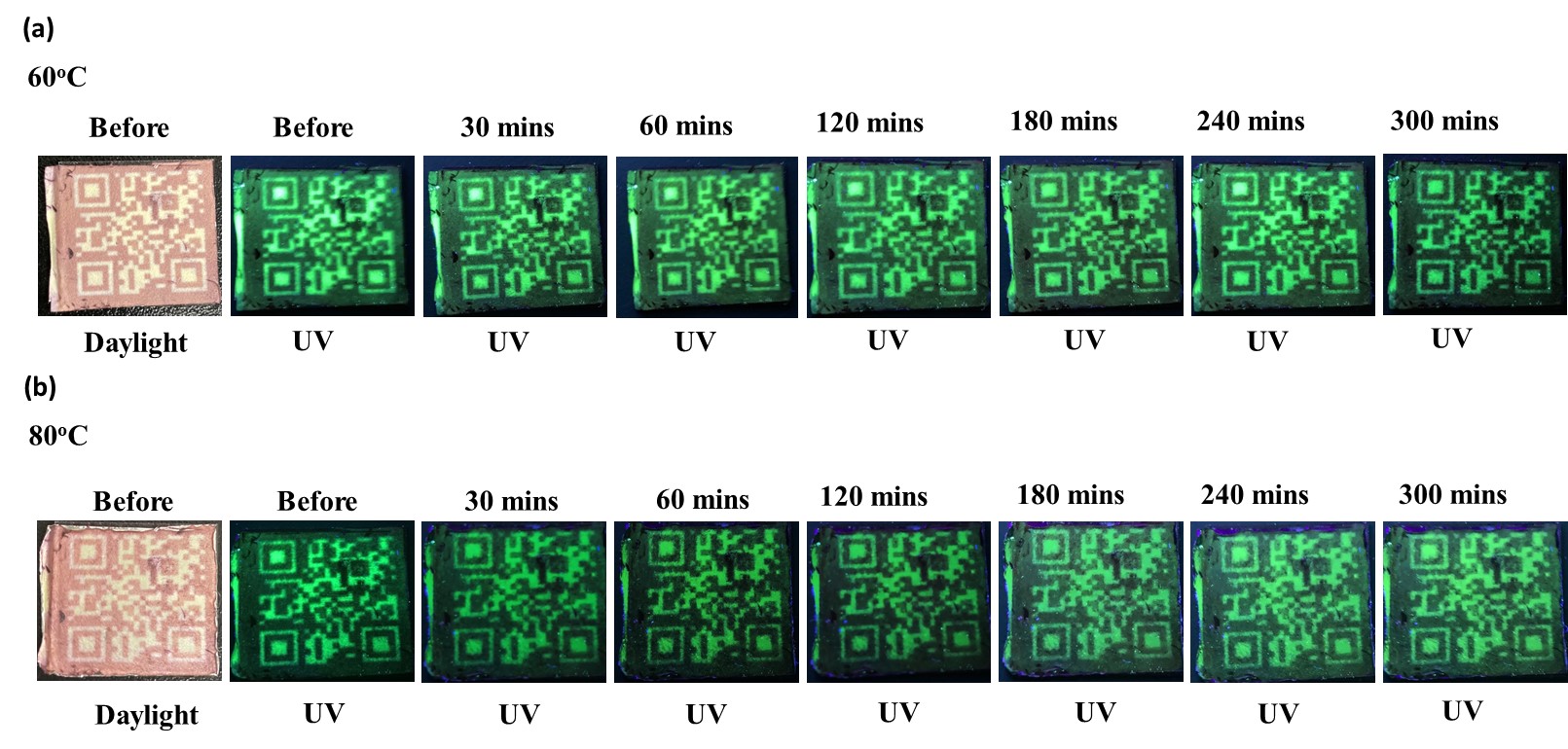


**Fig. S10:** Photographs of the QR code patterns printed on PCL-perovskite fibers-based paper under daylight and UV light (365 nm) at (a) 60°C and (b) 80°C at different time interval.


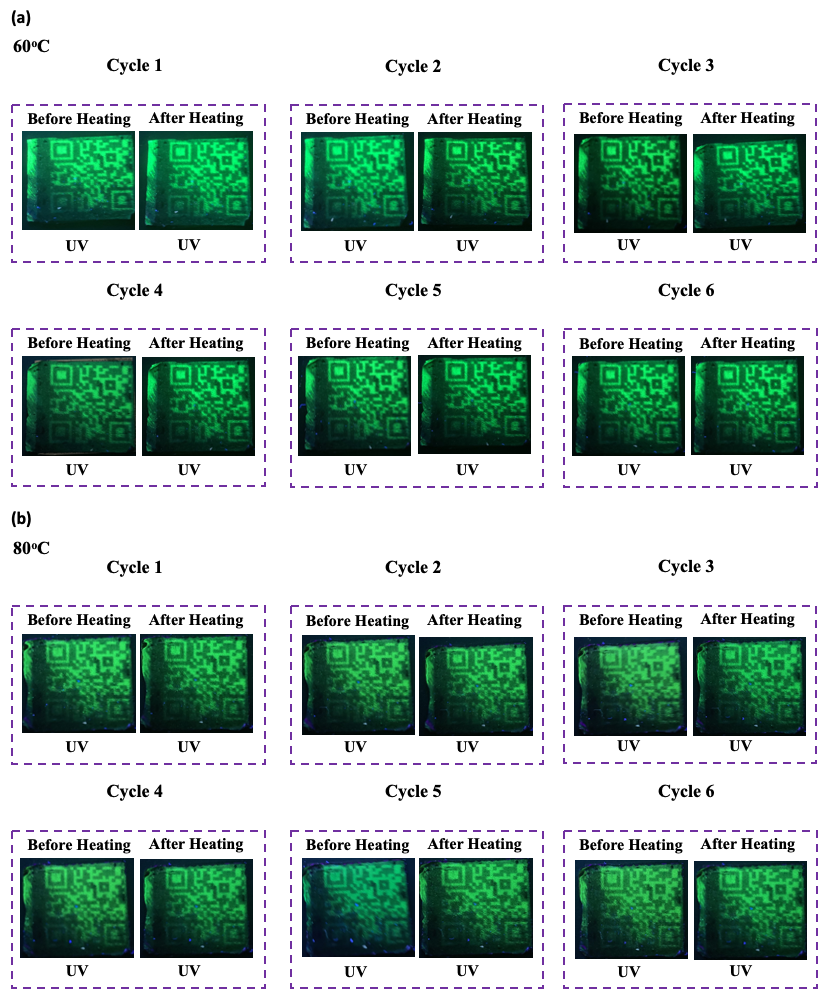


**Fig. S11:** Thermal stability heating cycles: photographs of the QR code printed on PCL-perovskite fibers-based paper, taken before and after heating at different cycle under UV light (365 nm) at (a) 60°C and
(b) 80°C.
